# From Sequential Gland Replacement to Recurrent Gland Coordination: A Comparative Framework for Subventral and Dorsal Oesophageal Gland Effectors Across Plant-Parasitic Nematode Lifestyles

**DOI:** 10.3390/plants15172623

**Published:** 2026-08-27

**Authors:** Phatu William Mashela, Kgabo Martha Pofu

**Affiliations:** Department of Plant Production, Soil Science and Agricultural Engineering, Green Biotechnologies Research Centre of Excellence, University of Limpopo, Sovenga 0727, South Africa

**Keywords:** dorsal gland, subventral glands, gland-role transition, recurrent gland coordination, stylet-secreted effectors, parasitic lifestyle, feeding-site maintenance, plant-parasitic nematodes

## Abstract

Plant-parasitic nematodes manipulate host tissues through stylet-secreted gene products synthesised principally in two subventral and one dorsal oesophageal gland. Earlier reviews have catalogued effector repertoires, described feeding-site formation, and explained how individual effectors modify host defence, development, and metabolism. However, the temporal coordination of the gland cells themselves has not been comparatively synthesised across parasitic lifestyles. This review therefore advances a gland-centred, lifestyle-dependent framework. In sedentary endoparasites, available evidence supports a pronounced developmental transition: subventral gland products dominate penetration and migration, whereas dorsal gland products become increasingly important during feeding-site initiation and maintenance. Migratory endoparasites repeatedly penetrate, migrate, and feed without establishing permanent feeding cells; their gland activity is consequently predicted to be recurrent and overlapping rather than a one-way replacement. Ectoparasites likewise require behaviour-dependent coordination during repeated probing and external feeding, although direct gland localisation evidence remains limited. We integrate gland origin, secretion chemistry, infection stage, and parasitic behaviour across root-knot, cyst, citrus, false root-knot, lesion, burrowing, and ectoparasitic nematodes. The synthesis distinguishes experimentally demonstrated gland localisation from evidence-weighted inference and formulates testable predictions for comparative gland transcriptomics, spatial expression, and functional silencing. This framework also identifies gland activation, secretion, and stage-critical products as targets for RNA interference, genome editing, resistance breeding, and sustainable nematode management. The principal novelty is therefore not another catalogue of nematode effectors, but a comparative model explaining when and why subventral and dorsal glands exchange, retain, or alternate their functions across contrasting parasitic lifestyles.

## 1. Introduction

Plant-parasitic nematodes represent one of the most biologically specialised groups of plant pathogens. Their success is rooted in their ability to penetrate plant tissues with a stylet and inject secretions that alter plant cells before and during feeding. In the framework used here, these nematodes include sedentary endoparasites, migratory endoparasites and ectoparasites; these functional categories are widely used in plant nematology because they reflect fundamentally different feeding behaviours, tissue interactions and disease outcomes [1,2,3].

Across these groups, parasitism depends on secretions produced in the oesophageal glands. Nematode effectors are synthesised in specialised oesophageal gland cells and secreted through the stylet into plant tissues, where they facilitate invasion, suppress defence responses, manipulate host development, and support feeding-site establishment or short-term feeding depending on nematode lifestyle [4,5,6,7].

The word “chemicals” is sometimes used to describe these gland products. However, in molecular plant nematology, the more accurate terms are “effector gene products”, “parasitism proteins”, “secreted effectors”, or “stylet-secreted proteins”. Most are proteinaceous products encoded by parasitism genes, although their biological effects include enzymatic degradation, peptide mimicry, hormone disruption, immune suppression, nuclear reprogramming, and metabolic redirection [8,9,10].

### 1.1. Novelty and Contribution of This Review

Previous syntheses have organised plant-parasitic nematode biology principally around effector identity, molecular function, evolution, or the host pathways manipulated. Gheysen and Mitchum [11] examined the manipulation of plant developmental pathways; Hewezi and Baum [12] and Mitchum et al. [8] concentrated on stylet-secreted effectors of cyst and root-knot nematodes and their roles in host-cell reprogramming; Kyndt et al. [13] reviewed the specialised feeding organs induced in roots; and Jones et al. [1] situated the most economically important nematodes within molecular plant pathology. Vieira and Gleason [9] synthesised effector diversity and discovery tools, whereas Espada et al. [14] demonstrated a multilayered detoxification strategy in the migratory pinewood nematode (*Bursaphelenchus xylophilus*). Collectively, these publications define the effector-centred foundation on which the present synthesis builds.

The distinctive contribution of this review is that its primary unit of analysis is the oesophageal gland system rather than the individual effector or manipulated host pathway. It integrates gland identity, secretion chemistry, infection stage, and parasitic behaviour to ask when the two subventral glands and the single dorsal gland become active, how their relative contributions change during parasitism, and whether the widely cited transition from subventral- to dorsal gland dominance is universal.

The central hypothesis is that a pronounced, directional subventral-to-dorsal transition is primarily a specialisation of sedentary endoparasitism. Root-knot and cyst nematodes shift from mobile invasion to permanent feeding-site establishment, creating a biological basis for early subventral gland dominance and later dorsal gland dominance. By contrast, migratory endoparasites repeatedly penetrate, migrate, and feed, while ectoparasites repeatedly probe and feed from outside the root. These lifestyles predict recurrent, overlapping, or alternating gland deployment rather than permanent replacement of one gland system by the other.

A further innovation is the explicit grading of evidence. Gland-specific expression and temporal switching are comparatively well supported in root-knot and cyst nematodes but remain fragmented for migratory endoparasites and ectoparasites. The review therefore separates experimentally localised secretion from functional inference and converts the proposed lifestyle differences into testable predictions for single-gland transcriptomics, spatial localisation, time-resolved secretomics, and gene silencing.

Accordingly, the review does not duplicate earlier effector catalogues or accounts of feeding-site development. It offers a comparative theory of gland deployment and identifies gland activation, coordination, secretion, and stage-critical products as intervention points for host-induced gene silencing, RNA interference, genome editing, resistance breeding, and sustainable nematode management.

### 1.2. Literature Search and Review Strategy

This review was developed as a narrative, evidence synthesis review anchored in three nematode lifestyle classes that are central to plant nematology: sedentary endoparasites, migratory endoparasites, and ectoparasites. The literature search prioritised peer-reviewed reviews and primary studies on oesophageal or pharyngeal gland gene products, stylet-secreted effectors, feeding-site induction, defence suppression, transgenic resistance, host-delivered RNA interference, and small-RNA biology in plant-parasitic nematodes. The principal search concepts were combined using terms such as plant-parasitic nematode effectors, oesophageal gland, dorsal gland, subventral gland, stylet secretion, *Meloidogyne effectors*, cyst nematode CLE, SPRYSEC, *Pratylenchus* effector, *Radopholus* effector, *Tylenchulus semipenetrans*, *Nacobbus aberrans*, HIGS, HD-RNAi, CRISPR/Cas9, nematode-responsive promoter, and nematode microRNA.

The review gave the highest weight to studies that included gland localisation, transcriptomic or gland-cell expression evidence, functional validation, transgenic plant assays, host target identification, or clear parasitological phenotypes. Broad effector reviews were used to frame the general biology of root-knot and cyst nematodes, while newer work on migratory endoparasites, effectoromes, nematode-responsive promoters, and small RNAs was used to update the more rapidly developing sections [3,6,7,15,16,17,18,19].

Because effector biology is unevenly developed across nematode groups, the review explicitly distinguishes between well-supported examples, such as named root-knot and cyst nematode effectors, and evidence-weighted inferences for under-studied groups such as *Tylenchulus*, *Nacobbus*, and several ectoparasites. This avoids overgeneralising from Meloidogyne, *Heterodera*, and *Globodera* to all plant-parasitic nematodes while still allowing comparative interpretation across lifestyles.

## 2. Anatomical and Functional Basis of Oesophageal Gland Secretion

### 2.1. Developmental Coordination of Oesophageal Gland Activity in Sedentary Nematodes

The oesophageal glands are among the most important secretory organs in plant-parasitic nematodes. In sedentary endoparasites, the two subventral glands are strongly associated with penetration, migration, and early host modification, whereas the dorsal gland becomes increasingly active during feeding-site initiation and maintenance. This is best regarded as a change in relative dominance, not proof that the subventral glands become completely inactive. Differential gland activity has been demonstrated most clearly in root-knot and cyst nematodes, where stylet-secreted products mediate the transition from mobile invasion to permanent host dependence [4,8,12,16,20,21].

The stylet acts as both a mechanical puncturing structure and a molecular injection device. Through the stylet, the nematode releases gland products into the apoplast, plant cell wall, cytoplasm, nucleus, or nucleolus. This stylet-based delivery system is essential because feeding-site formation and host compatibility depend not only on physical penetration but also on the accurate delivery of effectors into specific host-cell compartments [6,15,20].

This temporal and spatial targeting is one reason nematode parasitism is highly efficient. The nematode releases the right type of molecule at the right stage of infection. Early secretions promote invasion and migration, while later secretions sustain feeding cells, suppress defence, and modify nutrient flow. This staged deployment is consistent with gland-cell transcriptomic studies and with functional analyses of effectors expressed during different parasitic phases [4,21,22].

**Figure 1:**
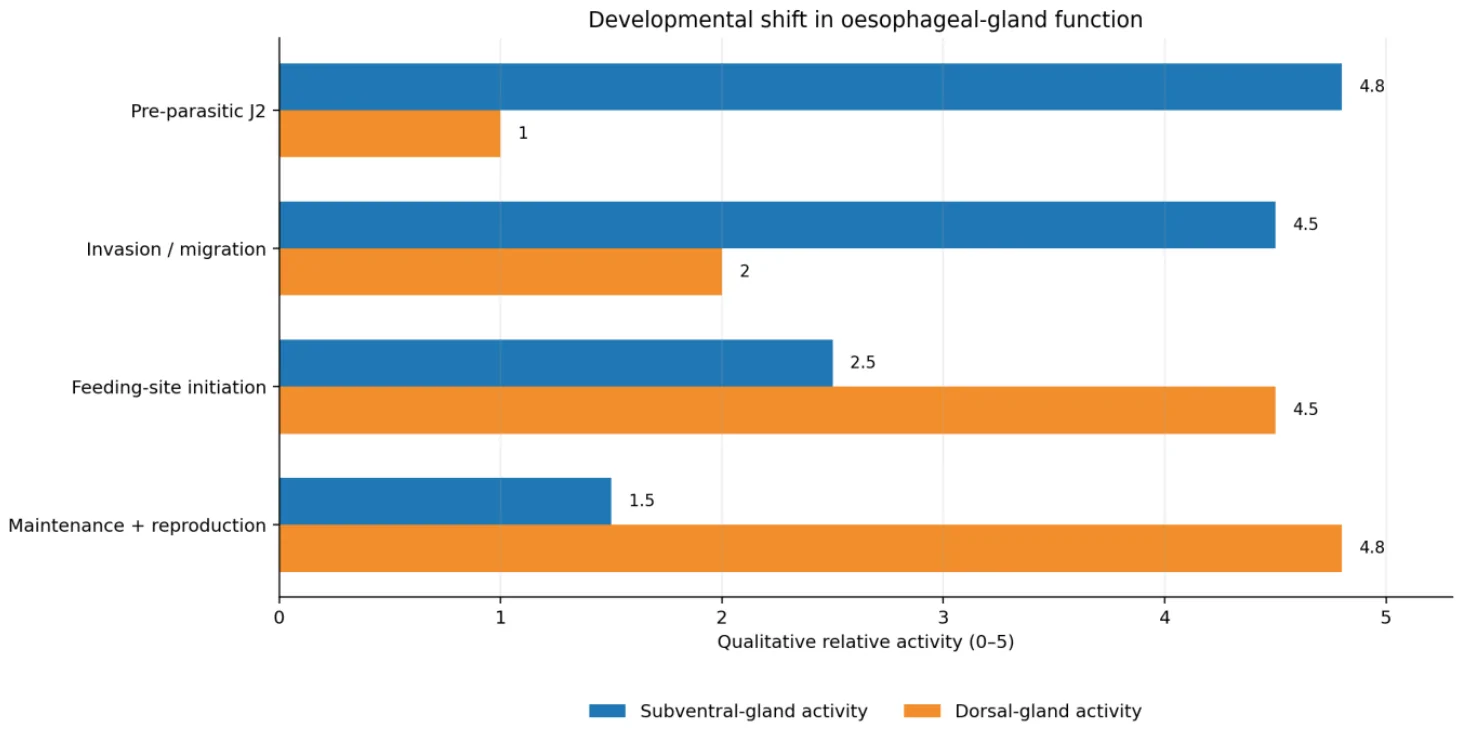
Comparative bar chart showing the literature-derived developmental shift from predominant subventral gland activity during pre-parasitic and migratory stages to predominant dorsal gland activity during feeding-site initiation and maintenance. Values are qualitative synthesis scores (0–5), not experimental measurements [4,16,20].

The frequently cited subventral-to-dorsal gland transition is the reference pattern against which other lifestyles should be compared. In sedentary endoparasites, infective juveniles rely heavily on subventral gland products during penetration and migration to the vascular cylinder; following feeding-site initiation, dorsal gland transcription and secretion become increasingly prominent. The biological transition is therefore from transient tissue invasion to long-term manipulation of giant cells or syncytia (Figure 1). Earlier reviews established the importance of effector-mediated host reprogramming and feeding-site development [8,11,12,13] but did not use the temporal relationship between the three gland cells as a comparative framework across sedentary, migratory, and ectoparasitic lifestyles.

### 2.2. Functional Coordination of Gland Activity in Migratory and Ectoparasitic Nematodes

In migratory endoparasites, such as *Pratylenchus*, *Radopholus*, *Ditylenchus*, and *Bursaphelenchus*, an equivalent permanent exchange is biologically unlikely because no permanent feeding site is established. These nematodes repeatedly penetrate cells, migrate through tissues, feed briefly, and move again. Cell wall degradation and multilayered detoxification are therefore repeatedly required, as illustrated for *B. xylophilus* [14]. We hypothesise that subventral gland products remain important throughout infection for renewed penetration and migration, while dorsal gland products contribute during successive episodes of feeding, local host-cell modulation, and defence suppression. The gland system should consequently display recurrent cycles, partial overlap, or rapid alternation rather than a one-way developmental replacement [3,23]. Developmental shift in oesophageal function is summarised on Figure 1.

In ectoparasitic nematodes, including many longidorids, trichodorids, and other externally feeding taxa, the relationship is also feeding cycle-dependent. These nematodes remain outside the root and insert the stylet into epidermal, cortical, or deeper cells without internal migration and without establishing permanent feeding cells. Subventral gland secretions are expected to support probing, stylet penetration, and local wall or apoplastic modification, whereas dorsal gland products are likely to contribute to host-cell conditioning, sustained nutrient withdrawal, and local defence modulation during feeding. However, because ectoparasite effector biology is less well resolved than that of root-knot and cyst nematodes, these assignments should be treated as evidence-weighted functional inferences rather than universally demonstrated rules [2,7,24].

Thus, the classic gland-role exchange should not be generalised to all plant-parasitic nematodes. It is most appropriate for sedentary endoparasites that switch from migration to permanent feeding-site maintenance. In mobile endoparasites and ectoparasites, subventral and dorsal glands function in overlapping, recurrent, and behaviour-dependent patterns governed by repeated penetration, probing, temporary feeding, and movement.

## 3. Chemical Nature and Major Classes of Gland-Derived Gene Products

The products released from oesophageal glands are diverse. They can be grouped into several functional classes, although many effectors have multiple roles. Table 1 summarises the major effector classes recognised in the literature, including cell wall-degrading enzymes, developmental peptide mimics, defence suppressors, nuclear-targeted effectors, metabolic reprogrammers, and feeding-site maintenance proteins [5,6,7,8].

### 3.1. Cell Wall-Degrading and Cell Wall-Modifying Enzymes

Plant cell walls are major physical barriers to nematode invasion. Cell wall-degrading enzymes such as beta-1,4-endoglucanases, pectate lyases, polygalacturonases, and pectin methylesterases allow nematodes to soften, loosen, or partially degrade cell walls during root penetration, migration, and feeding-site expansion. These enzymes are important in both sedentary endoparasites and migratory endoparasites, although their functional emphasis differs between feeding-site establishment and destructive migration [3,5,25,26].

### 3.2. Peptide Hormone Mimics and Developmental Regulators

One of the most elegant features of nematode parasitism is molecular mimicry. Cyst nematodes secrete CLE-like peptides that mimic plant developmental peptides and promote syncytium formation. More recent work has expanded nematode mimicry to other peptide classes, including CEP-, RALF-, IDA-, and PSY-like effectors, showing that nematodes can hijack host developmental signalling to redirect plant growth towards feeding-site formation [27,28,29].

### 3.3. Defence-Suppressing Proteins

Over time, plants respond to nematode invasion through reactive oxygen species, cell wall reinforcement, salicylic acid, and jasmonic acid pathways, hypersensitive responses, and localised defence gene activation. Defence-suppressing effectors interfere with these responses and prevent the nematode from maintaining living host cells long enough for feeding and reproduction. Examples include calreticulins, SPRYSEC proteins, venom allergen-like proteins, glutathione-S-transferases, peroxiredoxins, and fatty-acid/retinol-binding proteins [6,7,15,30].

### 3.4. Nuclear-Targeted and Transcription-Modifying Effectors

Some secreted effectors are transported into the host nucleus or nucleolus. These effectors are especially important because feeding-site formation requires deep reprogramming of host gene expression, cell-cycle regulation, and plant developmental identity. Nuclear-targeted effectors such as MiEFF1 and other characterised sedentary nematode effectors demonstrate that nematodes can manipulate host transcriptional and regulatory processes from within plant cells [15,16,31].

### 3.5. Metabolic Reprogrammers and Nutrient Sink Effectors

Feeding sites function as nutrient sinks. Their establishment requires reallocation of carbon, nitrogen, minerals, and water from normal plant growth to nematode feeding. Several effectors influence transport, hormone signalling, cytoskeletal organisation, cell expansion, and host metabolism. The development- and physiology-altering capacity of nematode effectors is now recognised as a central but still under-explored component of parasitism [6,8,10].

### 3.6. Effector-by-Genus Evidence Map

The strength of evidence for specific gland-derived gene products differs greatly among genera. Root-knot and cyst nematodes have the richest functional literature, including named effectors, host targets, and transgenic or RNAi validation. Migratory endoparasites are increasingly represented through transcriptomic and effector-candidate studies, whereas the semi-endoparasites *Tylenchulus* and Nacobbus, along with many ectoparasites, remain more dependent on comparative inference and emerging genomic resources [1,3,7,9,17]. Table 2 summarises representative and readily available evidence without implying that the examples are exhaustive.

## 4. Roles in Sedentary Endoparasitic Nematodes

Sedentary endoparasites make the most sophisticated use of oesophageal gland products because they depend on permanent or semi-permanent feeding structures. This group includes root-knot nematodes, cyst nematodes, the citrus nematode, and false root-knot nematodes. Their effectors transform ordinary root cells into giant cells, syncytia, nurse cells, or specialised feeding structures that remain alive and metabolically active for prolonged periods [2,15,16].

### 4.1. Root-Knot Nematodes: Meloidogyne Species

Root-knot nematodes induce giant cells. Infective second-stage juveniles enter the root, migrate towards vascular tissues, and establish permanent feeding sites by reprogramming selected cells into enlarged, multinucleate giant cells. The surrounding tissues proliferate, producing the visible root galls that are characteristic of *Meloidogyne* infection. The process depends on a coordinated effector arsenal that acts during invasion, feeding-cell induction, and feeding-cell maintenance [15,16,20].

The parasitic role of *Meloidogyne* effectors can be summarised as invasion, immune suppression, cell-cycle disruption, metabolic reprogramming, and feeding-site maintenance. Cell wall enzymes facilitate early movement, defence suppressors protect the nematode from host responses, and nuclear or cytoplasmic effectors redirect host-cell identity. Dorsal gland effectors discovered from adult female stages further show that parasitism continues to require effector delivery after feeding sites have already been established [21,22,33].

### 4.2. Cyst Nematodes: Heterodera and Globodera

Cyst nematodes induce syncytia. Unlike giant cells, syncytia form when cell walls between adjacent cells partially dissolve, allowing many cells to merge into one large feeding structure with multi-cellular organelles. Oesophageal gland products are central to this process because they suppress defence, remodel cell walls, alter developmental signalling, and maintain the syncytium as a nutrient source for the sedentary female [2,8,15].

Effector diversity in cyst nematodes is especially important for host specificity and virulence. Variation in effector repertoires can help populations overcome resistance genes or adapt to new host genotypes. Examples include variable SPRYSEC proteins, CLE-like peptide mimics, and effectors that interact with host signalling or immune components [27,32,34].

### 4.3. Citrus Nematode: Tylenchulus semipenetrans

*Tylenchulus semipenetrans* is a sedentary semi-endoparasite. Juveniles and young females may feed ectoparasitically, but mature females establish a sedentary relationship in which the anterior region is embedded in root tissues while the posterior body remains outside. The nematode establishes nurse cells that provide nutrients to developing females and is associated with citrus slow decline, reduced root function, and reduced fruit yield [2,35,36].

Compared with *Meloidogyne* and cyst nematodes, the molecular effector biology of *T. semipenetrans* remains underdeveloped. Nevertheless, the biology of nurse-cell formation strongly implies a requirement for gland-derived effectors that suppress defence, maintain living cortical cells, and redirect nutrient flow. The limited molecular information available for this genus is therefore a major gap in effector biology and citrus nematode management [2,36].

### 4.4. False Root-Knot Nematodes: Nacobbus Species

*Nacobbus species* occupy an unusual evolutionary and biological position. *Nacobbus aberrans* is recognised for having both migratory endoparasitic and sedentary endoparasitic stages. Its sedentary stage has features that resemble both root-knot and cyst nematodes, and transcriptomic analysis indicates that its effector complement provides insight into the evolution of sedentary endoparasitism [2]. 

The role of gland products in *Nacobbus* is therefore dual. Early effectors support invasion and migration, while later effectors support feeding-site formation. This dual parasitic strategy makes *Nacobbus* an important bridge between migratory and sedentary nematode biology and a useful model for understanding how different parasitic lifestyles may have evolved [2].

## 5. Roles in Migratory *Endoparasitic nematodes*

Migratory endoparasites differ from sedentary endoparasites because they do not establish permanent feeding sites. Instead, they enter roots, migrate through tissues, feed from multiple cells, and produce lesions or necrosis. Their gland-derived gene products therefore support repeated penetration, cell wall degradation, detoxification, defence suppression, and short-term feeding, rather than long-term feeding-cell maintenance [3,23].

### 5.1. Root-Lesion Nematodes: Pratylenchus Species

*Pratylenchus* species are migratory endoparasites that feed and reproduce within root tissues. Their gland-derived products support penetration, cell wall modification, host defence suppression, and repeated feeding as they move through cortical tissues. The visible outcomes are lesion formation, tissue necrosis, and increased vulnerability to secondary pathogens, especially fungi and bacteria [1,3,23].

### 5.2. Burrowing Nematodes: Radopholus similis and Radopholus citrophilus

*Radopholus* spp. are destructive migratory endoparasites of banana, citrus, and other crops. They penetrate roots, migrate through cortical tissues, and cause extensive root necrosis. Effector candidates, including cell wall-modifying enzymes such as pectate lyases, support invasion and migration, consistent with a parasitic strategy based on repeated tissue destruction rather than permanent feeding-site development [1,3,23]. The expected gland use pattern is therefore recurrent rather than stage-replacement-based, with subventral gland functions supporting repeated burrowing and cortical migration, and dorsal gland products contributing during temporary feeding and local defence modulation.

## 6. Roles in Ectoparasitic Nematodes

Ectoparasitic nematodes remain outside the root while inserting the stylet into epidermal, cortical, or deeper cells. Their oesophageal gland products condition plant cells before ingestion and can cause localised cell damage, root-tip swelling, stubby-root symptoms, or reduced root growth. Although their effectors are less studied than those of sedentary endoparasites, ectoparasites also depend on stylet-mediated modification of living host cells before nutrient withdrawal [2,24].

Some ectoparasitic nematodes are important virus vectors. *Xiphinema* and *Longidorus species* transmit nepoviruses, while *Trichodorus* and *Paratrichodorus* transmit tobraviruses. Virus transmission is not simply a chemical function of gland secretions; it depends on feeding behaviour, stylet and odontophore structures, virus retention sites, and highly specific interactions between nematode vectors and virus particles [24,37,38]. In these ectoparasitic systems, gland use should likewise be interpreted as probing and feeding cycle-dependent, with subventral gland secretions assisting stylet penetration and local conditioning, and dorsal gland products probably contributing during sustained feeding from externally accessed cells.

## 7. Comparative Synthesis Across Nematode Lifestyles

**Figure 2:**
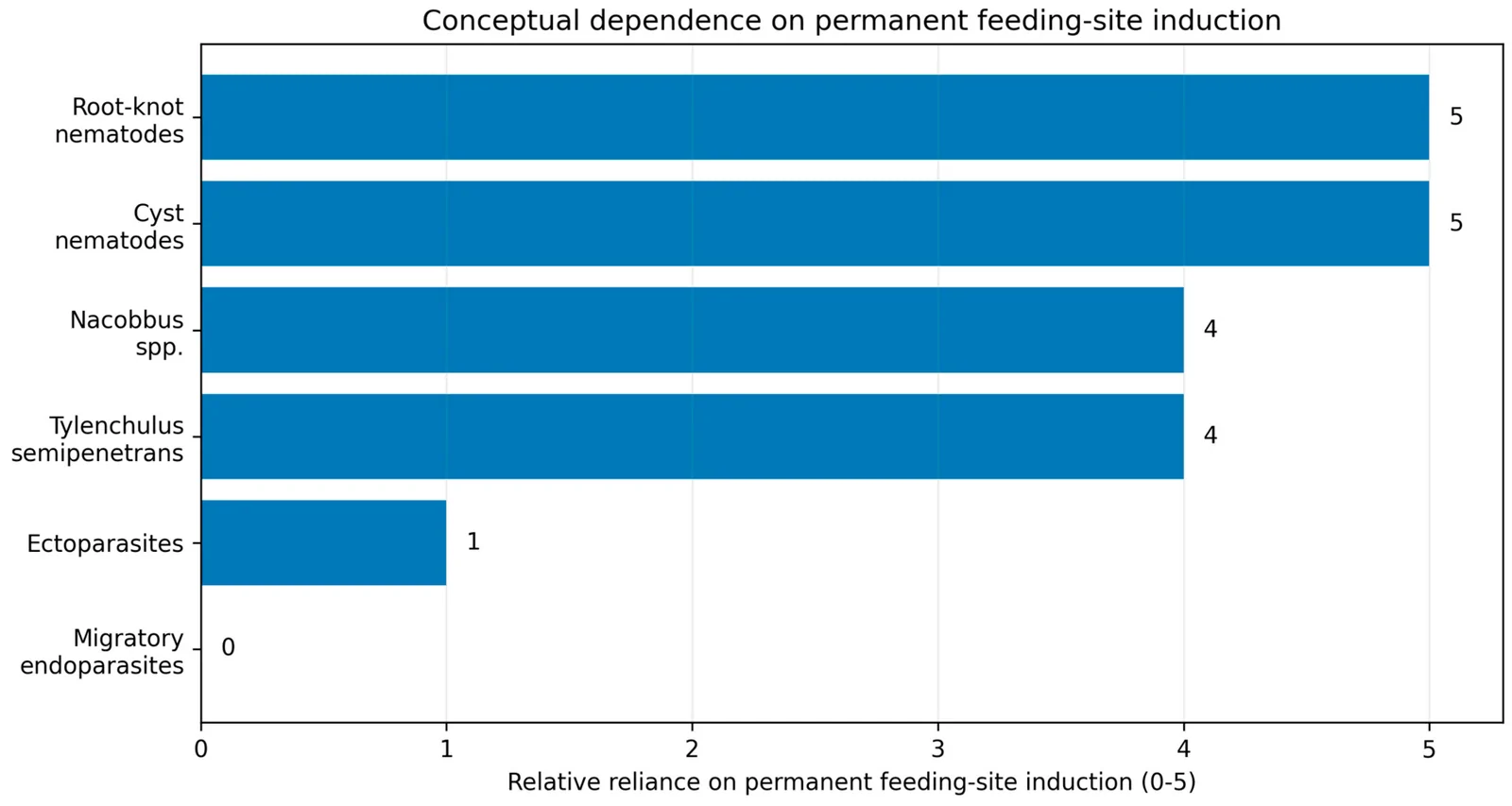
Conceptual dependence of major plant-parasitic nematode groups on permanent feeding-site induction. The scale is qualitative and is intended for synthesis, not as raw experimental data. Interpretive scores are based on the feeding-site biology described for root-knot, cyst, citrus, false root-knot, migratory, and ectoparasitic nematodes [2,3,23].

**Figure 3:**
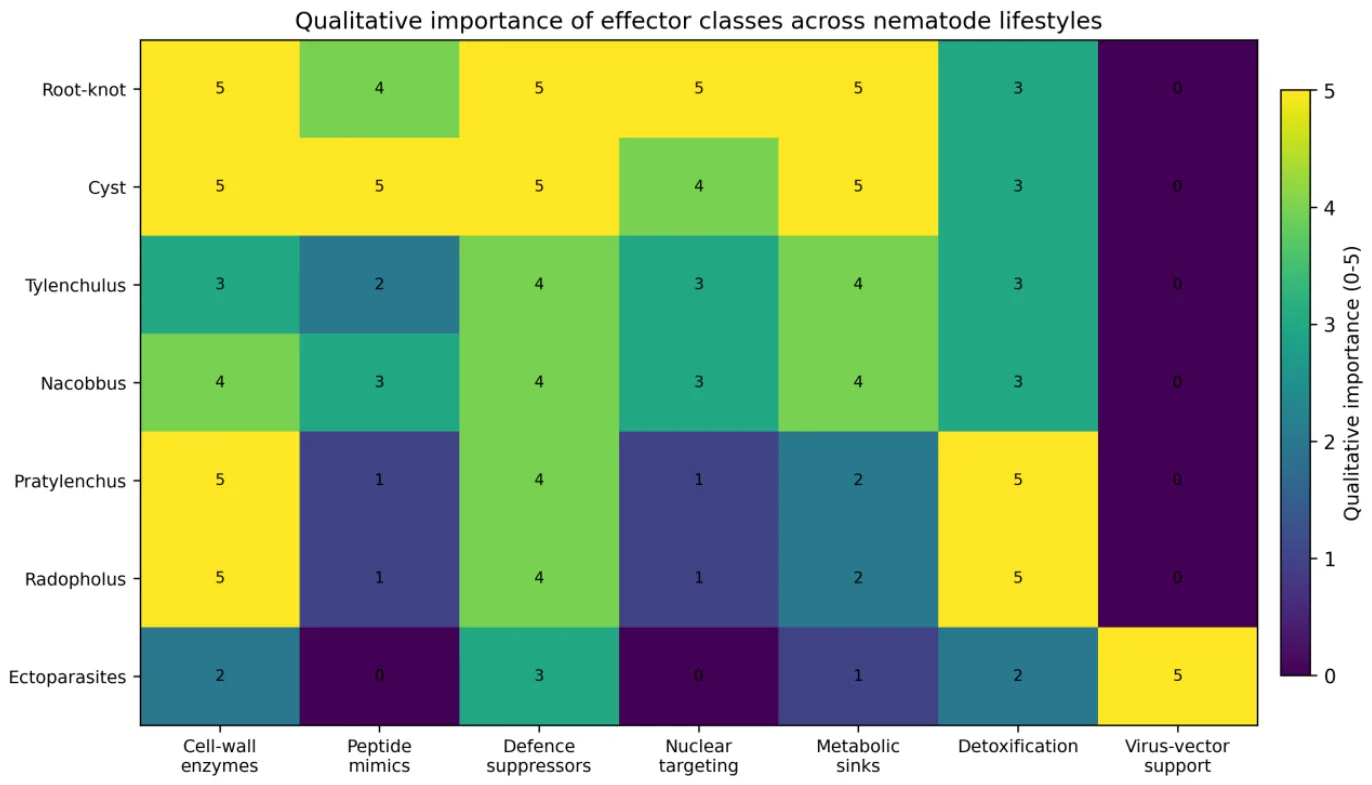
Qualitative effector-function matrix showing relative importance of selected effector classes across plant-parasitic nematode groups. Values are interpretive scores from 0 to 5 based on literature synthesis and should not be treated as experimental measurements [5,6,7].

**Figure 4:**
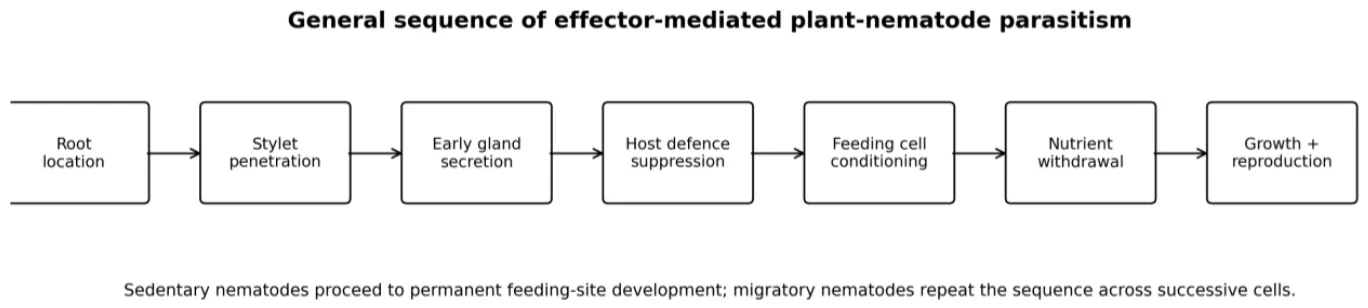
General sequence of effector-mediated parasitism. Sedentary nematodes proceed to permanent feeding-site development, whereas migratory nematodes repeat the process across successive cells. The sequence reflects published models of stylet-secreted effector delivery and feeding-site or lesion development [2,3,15].

Comparatively, oesophageal gland-derived products show conserved biochemical functions but contrasting temporal organisation. Sedentary endoparasites undergo the clearest transition from invasion-associated subventral activity to dorsal gland-supported feeding-site maintenance. Migratory endoparasites are predicted to reactivate penetration-, detoxification-, and feeding-associated programmes in recurrent cycles, whereas ectoparasites probably coordinate gland output with probing duration, stylet position, and repeated access to host cells. This lifestyle-dependent gland coordination model extends earlier accounts of effector diversity, host manipulation, and feeding-site biology [1,8,9,11,12,13] by explaining how gland deployment itself may be reorganised by parasitic behaviour. Figure 2, Figure 3 and Figure 4 and Table 3 each summarises comparative synthesis across nematode lifestyles.

## 8. Transgenic Plant Literature, RNAi, HIGS, and Genome Editing as Functional Validation of Effector Biology

The literature on transgenic plants has substantially promoted the subject reviewed here because it has provided functional evidence that oesophageal gland-derived gene products are not merely associated with parasitism but are often required for it. Classical microscopy and transcriptomics revealed gland activity and candidate effectors; transgenic plant experiments then tested whether manipulating either the host or the nematode target gene could interrupt invasion, feeding-site establishment, or reproduction. This is why transgenic resistance studies are now central to effector biology, especially for root-knot and cyst nematodes [25,26,39,40].

Transgenic approaches have contributed to this subject in five major ways. First, the transfer or expression of natural resistance genes, such as Mi-1.2 in tomato, demonstrated that host resistance can recognise, or block nematode parasitism processes associated with effector delivery. Second, expression of proteinase inhibitors and cystatins showed that plant-produced anti-feedant proteins can interfere with nematode nutrition after stylet feeding has begun. Third, chemodisruptive peptides and root-specific expression systems demonstrated that transgenic products could reduce root invasion without direct broad-spectrum toxicity. Fourth, host-delivered RNA interference, or HD-RNAi, provided direct evidence that nematode parasitism genes can be silenced from within the plant host. Fifth, nematode-responsive promoters have refined the approach by restricting anti-nematode expression to infection sites, thereby linking biotechnology to the spatial biology of feeding-site induction [18,41,42].

### 8.1. Host-Delivered RNA Interference as Proof of Effector Essentiality

HD-RNAi is the most direct transgenic bridge between oesophageal gland biology and nematode control. In this approach, the plant expresses double-stranded RNA corresponding to an essential nematode gene. When the nematode feeds, it takes up RNA molecules that trigger sequence-specific degradation of the nematode transcript. This approach is especially powerful for effector genes because it tests whether a gland-derived product is necessary for parasitism. The classic example is the root-knot nematode 16D10 parasitism gene: transgenic *Arabidopsis* expressing 16D10 dsRNA showed broad resistance against major *Meloidogyne species*, establishing that a conserved secreted parasitism gene could be targeted from the host plant [43].

Subsequent HD-RNAi studies strengthened this principle. *Arabidopsis lines* expressing dsRNA against the *Meloidogyne incognita* effector Mi-msp2 showed reduced galls, females, and egg masses, demonstrating that silencing an effector gene compromises nematode development and reproduction [44]. Reviews of HD-RNAi have concluded that effector or secretory protein genes are among the most favourable RNAi targets because they are directly involved in the host–parasite relationship [40].

Host-delivered RNAi and host-induced gene silencing are especially relevant to this review because they convert effector biology into a functional test: if silencing a gland-derived transcript reduces galling, female development, egg production, or reproduction factor, the targeted gene product is likely to be biologically important for parasitism. In Arabidopsis, HD-RNAi-mediated silencing of the *Meloidogyne incognita* effector gene Mi-msp2 reduced galls, females, and egg masses, and reduced Mi-msp2 mRNA abundance in females feeding on transgenic plants [44]. Earlier host-delivered RNAi studies, including targeting *Meloidogyne parasitism* genes, established the principle that plants can deliver silencing triggers to feeding nematodes and thereby reduce infection [39,43,45].

The RNAi literature also introduces necessary caution. RNAi efficiency varies with gene target, nematode species, delivery route, developmental stage, and dsRNA processing. Therefore, effector-centred RNAi should be interpreted as both a resistance tool and a functional validation method, not as a universally reliable stand-alone management strategy [39,46].

### 8.2. Resistance Genes, Cystatins, and Chemodisruptive Peptides

Transgenic expression of natural nematode resistance genes has also advanced the subject by linking plant immunity to nematode parasitism mechanisms. The cloned tomato Mi-1.2 gene is sufficient to confer resistance to root-knot nematodes in susceptible tomato backgrounds, indicating that host recognition systems can block nematode infection before successful giant-cell establishment [47]. Similarly, cyst nematode resistance genes and related systems have helped define the interface between nematode effectors, host recognition, and feeding-site failure.

Proteinase inhibitors, especially cystatins, provide a complementary transgenic strategy. Rather than targeting effector secretion directly, they interfere with nematode digestion and nutrient acquisition after the nematode begins feeding. Transgenic potato studies against *Globodera pallida* showed that cystatin-based and peptide-based resistance traits can suppress potato cyst nematode parasitism without broad disruption of non-target soil nematode communities [42]. These studies are important because they show that transgenic approaches can target the biological consequences of stylet feeding without relying on conventional chemical nematicides.

### 8.3. Nematode-Responsive Promoters and Precise Expression at Infection Sites

A major limitation of early transgenic approaches was dependence on constitutive promoters, which may impose metabolic cost or express resistance products in tissues where they are not needed. Nematode-responsive promoters address this problem by driving expression specifically in roots, galls, syncytia, or infection sites. Recent reviews emphasise that nematode-inducible promoters can integrate with RNAi, CRISPR, and resistance constructs to achieve temporal and spatial precision in anti-nematode expression [18]. In the context of this review, such promoters are important because they mirror the biology of oesophageal gland secretion: both nematode effectors and plant defence constructs are most effective when expressed at the correct time and place during the parasitic sequence.

### 8.4. From RNAi to HIGS, HD-RNAi, and Spray-Induced Silencing

RNAi-based nematode control has progressed from soaking or in vitro gene-silencing assays to host-delivered systems in which the plant expresses dsRNA or hairpin constructs directed against nematode genes. In the context of oesophageal gland products, this has two advantages. First, it can test whether a candidate effector is required for successful infection. Second, it can convert knowledge of gland-secreted parasitism genes into a crop-protection strategy. HIGS has been reviewed as a major transgenic approach in plant nematology, with targets including parasitism genes, housekeeping genes, developmental genes, and genes involved in RNAi pathways themselves [39,40,46].

An emerging extension is spray-induced gene silencing or externally delivered dsRNA, which could reduce regulatory and public-acceptance barriers associated with stable transgenics. However, the stability, uptake, and delivery of dsRNA in soil and rhizosphere environments remain major challenges. Recent RNAi reviews therefore emphasise target selection, delivery efficiency, and environmental persistence as decisive factors in translating RNAi from molecular proof-of-concept into field-useful nematode management [46,48].

### 8.5. CRISPR/Cas Genome Editing and Susceptibility Gene Targeting

Genome editing provides a complementary route to effector-centred nematode resistance. Whereas RNAi often targets nematode transcripts directly, CRISPR/Cas approaches usually target plant susceptibility or compatibility genes that nematode effectors exploit during invasion, defence suppression or feeding-site formation. A review of CRISPR/Cas9 research in plant–nematode interactions concluded that targeted knockout of host susceptibility genes can improve nematode tolerance, although the technology has so far been used more often to investigate plant–nematode molecular biology than to deliver commercial nematode-resistant cultivars [49].

Direct experimental evidence is now emerging. CRISPR/Cas9-mediated mutagenesis of the rice susceptibility gene OsHPP04 enhanced resistance to the rice root-knot nematode *Meloidogyne graminicola*, and the edited rice plants showed enhanced immune responses without obvious growth defects in the evaluated lines [50]. More recently, CRISPR/Cas9-mediated knockout of cotton MLO3 was reported to reduce reproduction of *Rotylenchulus reniformis*, although the study also highlighted possible pleiotropic effects associated with resistance-related cell-death phenotypes [51]. Together, these studies show that genome editing can convert knowledge of host compatibility factors into durable resistance strategies, but each target must be evaluated carefully for agronomic side effects.

### 8.6. Relevance to the Present Review

**Figure 5:**
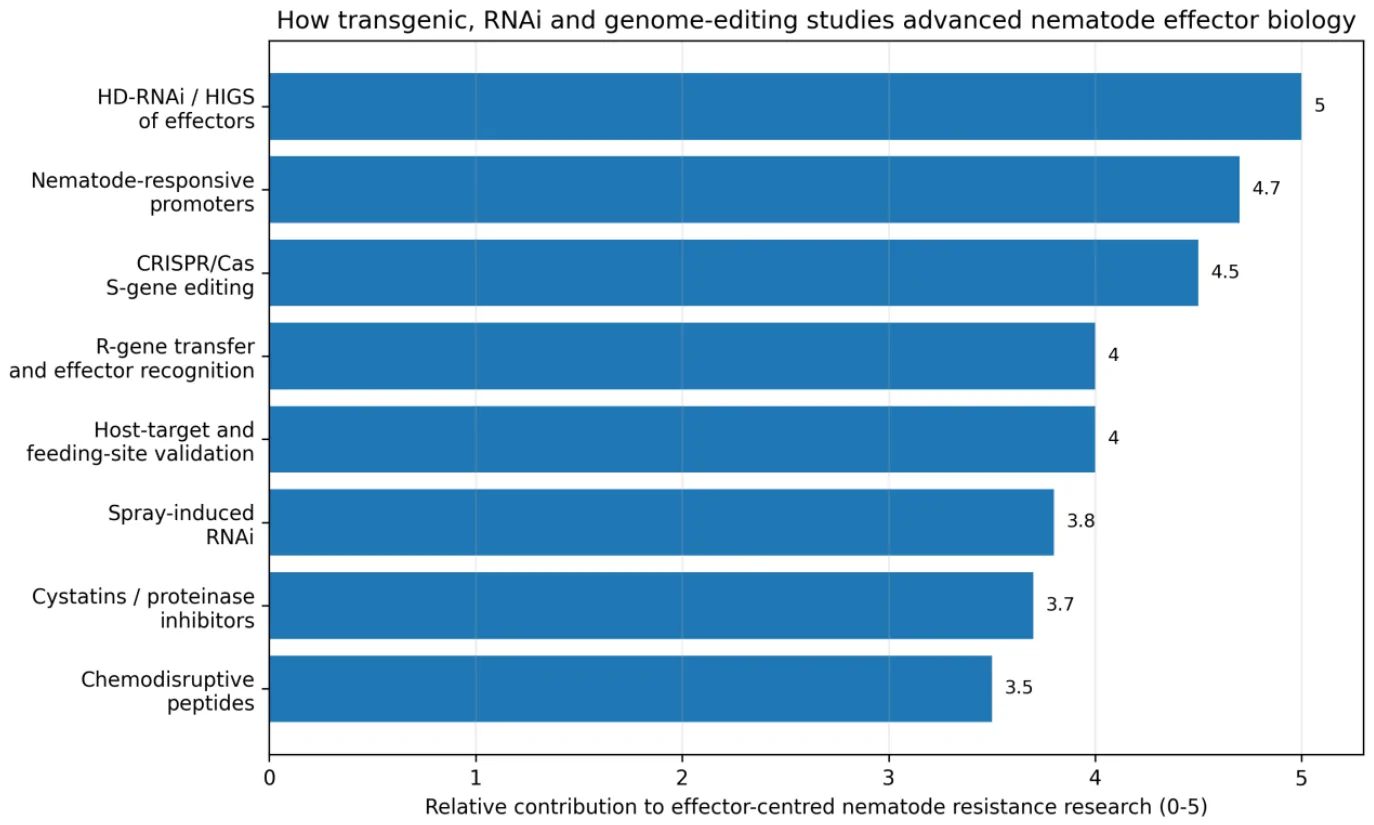
Conceptual synthesis of how transgenic, RNAi, HIGS, spray-induced RNAi, nematode-responsive promoter, and CRISPR/Cas studies have advanced effector-centred nematode resistance research. Scores are qualitative literature synthesis values and are not raw experimental measurements.

The transgenic literature therefore strengthens the central argument of this review. Oesophageal gland-derived gene products are central to parasitism because their suppression, recognition, or functional disruption reduces nematode infection (Figure 5; Table 4). Transgenic plants have promoted the field by converting effector biology from a descriptive discipline into a testable resistance platform. However, most successes remain concentrated in root-knot and cyst nematodes. Comparable transgenic validation is still limited for *Tylenchulus*, *Nacobbus*, *Pratylenchus*, *Radopholus*, and many ectoparasites, making these groups important priorities for future research.

## 9. Small RNAs and Cross-Kingdom Regulatory Signals

Most established oesophageal gland effectors are proteins or peptides, but the broader concept of nematode gene products now also includes small regulatory RNAs. MicroRNAs and other small RNAs regulate gene expression post-transcriptionally and are increasingly recognised as important in animal development, stress responses, and host–parasite interactions. For plant-parasitic nematodes, the small-RNA field remains younger than the protein-effector field, but it is developing quickly and may eventually explain aspects of parasitism that cannot be attributed solely to secreted enzymes or peptide mimics [19].

Small RNAs are important to include in this review for three reasons. First, nematode microRNAs may regulate the timing of parasitism genes, including those expressed during infective juvenile stages, migration, and feeding-site establishment. Second, RNAi studies demonstrate that biologically meaningful RNA movement can occur across the plant–nematode interface because plant-produced silencing triggers can reduce nematode gene expression in feeding nematodes [39,44,45]. Third, emerging cross-kingdom RNA literature raises the possibility that nematode-derived small RNAs could influence host transcripts, although this remains less fully demonstrated in plant-parasitic nematodes than in some fungal and oomycete systems [19,52].

The small-RNA perspective strengthens the central argument of the review: nematode parasitism is a gene-product-driven process. Protein effectors, peptide mimics, and enzymes are currently the best-supported secreted products of the oesophageal glands, but RNA-based regulation may modulate when, where, and how those effector genes act. Future research should therefore integrate gland-cell transcriptomics, small-RNA sequencing, host Argonaute-associated RNA profiling, and functional knockdown assays to determine whether small RNAs complement classical gland-derived effectors in host manipulation.

## 10. Management Implications: Diagnostic, Breeding, Biotechnology, and Integrated Nematode Management Value

Effector biology has practical implications for nematode management (Table 5). First, effectors can serve as diagnostic markers for identifying aggressive or virulent populations. Second, effector-informed breeding can identify cultivars that interrupt feeding-site formation. Third, RNA interference and host-induced gene silencing can target parasitism genes. Fourth, biological and botanical interventions can be evaluated by their ability to disrupt stylet activity, effector secretion, feeding-site establishment, or reproduction [7,9,39,41].

This shift is important for sustainable agriculture because nematode management should not be evaluated only through nematode mortality. A treatment may be valuable if it disrupts parasitism, even if it does not immediately kill the nematode. Effector-centred endpoints therefore complement conventional measures such as population density, reproduction factor, root galling, and yield loss, especially where sustainable or reduced-chemical nematode management is the goal [10,16,25,26].

### 10.1. Diagnostic and Surveillance Value

The diagnostic value of effector biology lies in its ability to move nematode detection beyond morphology and generic population counts. Effector genes and parasitism-associated markers can help distinguish closely related species, identify potentially virulent or resistance-breaking populations, and support pre-plant risk assessment. This is especially valuable where thermophilic *Meloidogyne species*, cyst nematodes, or mixed nematode communities occur in the same production system [1,7,9,55].

### 10.2. Resistance Breeding Value

For resistance breeding, effector biology provides a mechanistic bridge between phenotype and genotype. Instead of scoring only galling, cyst formation, or reproduction, breeders can ask whether a genotype prevents effector delivery, recognises an avirulence determinant, blocks a host target, or fails to support giant-cell, syncytium, or nurse-cell development. This strengthens the interpretation of host status and host sensitivity because the plant response is linked to a specific molecular stage of parasitism [10,16,34,47].

### 10.3. Biotechnological Value

The biotechnological value is most evident in RNAi, HIGS, HD-RNAi, spray-induced RNAi, nematode-responsive promoters, and CRISPR/Cas editing. These approaches have promoted the subject of this review because they functionally test whether a candidate gland product, parasitism gene, or host susceptibility factor is essential for infection. When silencing or editing reduces invasion, feeding-site establishment, female development, or egg production, the result provides functional evidence that the target is not merely expressed during parasitism but also contributes to parasitic success [18,39,43,44,49].

### 10.4. Integrated Nematode Management Value

For integrated nematode management, effector biology reframes control from nematode killing alone to interruption of the parasitic process. Resistant cultivars, crop rotation, biological control, botanicals, organic amendments, and induced resistance can be evaluated using endpoints such as reduced stylet activity, failed feeding-site induction, suppressed effector expression, impaired nutrient sink formation, and reduced reproduction. This broader framework is well aligned with sustainable agriculture because it supports reduced-chemical management and encourages integration of molecular, ecological, and agronomic tools [53,54,56].

## 11. Knowledge Gaps, Future Perspectives, and Priority Research Agenda

Despite major advances, the proposed gland coordination framework remains unevenly supported. Root-knot and cyst nematodes provide the strongest evidence for temporally differentiated gland activity, whereas *Tylenchulus*, *Nacobbus*, *Pratylenchus*, *Radopholus*, and most ectoparasites lack comparable time-resolved gland localisation data. The crucial unresolved question is no longer merely which effectors occur, but whether subventral and dorsal outputs switch, overlap, alternate, or reactivate during successive parasitic behaviours. Resolving this question will determine whether the classical sedentary model can be generalised or must be replaced by lifestyle-specific models [3,7,9].

Future research should prioritise paired single-gland transcriptomics, spatial transcript localisation, time-resolved secretomics, and functional silencing at defined behavioural stages: pre-penetration, migration, initial feeding, feeding-site initiation, and sustained feeding. Experiments should test whether subventral transcripts decline when dorsal transcripts rise in sedentary species, whether both programmes recur after each migration event in mobile endoparasites, and whether ectoparasite gland output changes with probing duration and feeding depth. These predictions make the proposed framework falsifiable rather than merely descriptive.

To convert effector biology into practical nematode management, the next phase of research should prioritise experimentally testable questions that connect gland-cell gene products to host response, field virulence, and control options. Table 6 summarises a proposed research agenda across the major nematode groups covered in this review.

## 12. Conclusions

The molecular basis of plant-parasitic nematode parasitism lies in the coordinated deployment of products from two subventral and one dorsal oesophageal gland. The synthesis developed here supports a pronounced change in relative gland dominance when sedentary endoparasites shift from invasion to permanent feeding-site maintenance, and predicts recurrent, overlapping, or behaviour-dependent gland activity in migratory endoparasites and ectoparasites. This distinction transforms gland biology from a descriptive anatomical observation into a comparative, testable model of parasitic adaptation. The framework therefore provides a focused basis for testing how lifestyle governs gland coordination and for identifying stage-specific vulnerabilities in plant–nematode interactions [3,4,8,9].

## Figures and Tables

**Table 1:** Major classes of oesophageal gland-derived gene products and their general roles in plant–nematode parasitism, synthesised from effector reviews and functional studies [5,6,7,8].

Effector class	Typical Chemical Nature	Examples	Primary Parasitic Role	Most Relevant Nematode Groups
Cell wall-degrading enzymes	Proteins/enzymes	Cellulases, pectate lyases, polygalacturonases, pectin methylesterases	Wall degradation, penetration, migration, and syncytium expansion	Sedentary and migratory endoparasites
Cell wall-modifying proteins	Proteins	Expansin-like proteins, cellulose-binding proteins	Cell wall loosening and feeding-site expansion	Root-knot and cyst nematodes
Plant peptide mimics	Peptides/proproteins	CLE-like, CEP-like, RALF-like, IDA-like, PSY-like, and PSK-like peptides	Mimic plant signalling and redirect development	Mainly sedentary endoparasites
Defence suppressors	Proteins/enzymes	Calreticulins, VAPs, SPRYSECs, GSTs, peroxiredoxins	Suppress immunity, ROS, and defence signalling	All major parasitic groups
Nuclear-targeted effectors	Secreted proteins/peptides	MiEFF1, Mi16D10, and related proteins	Alter transcription, root development, and cell-cycle pathways	Root-knot and cyst nematodes
Metabolic reprogrammers	Proteins/peptides	Solute transport, hormone, and cytoskeleton-associated effectors	Create and sustain nutrient sinks	Sedentary endoparasites
Detoxification and stress proteins	Enzymes and binding proteins	GSTs, antioxidant proteins, FAR proteins	Neutralise defence-related compounds	Migratory and sedentary nematodes
Feeding interface proteins	Secreted proteins	Feeding tube-associated secretions	Regulate nutrient uptake from host cells	Root-knot nematodes

**Table 2:** Effector-by-genus evidence map for oesophageal gland-derived gene products and related parasitism functions. Representative examples are synthesised from gland localisation, transcriptomic, and functional studies [3,7,22,32].

Genus or Group	Representative Gene Products or Effector Classes	Main Evidence Base	Role in Parasitism	Evidence Strength
*Meloidogyne* spp.	MiEFF1, MiEFF12, Mi16D10, Mi-msp2/Mi2G02, MiMSP32, RALF-like, and other secreted proteins	Gland expression, host localisation, host-target studies, overexpression, and HD-RNAi assays	Giant-cell induction, defence suppression, nuclear reprogramming, host-target manipulation, and reproduction support	High
*Heterodera* spp.	CLE-like peptides, 10A06, 4F01, chorismate mutases, cellulases, pectate lyases, SPRYSEC-like proteins	Gland-cell transcriptomics, in situ localisation, host-target studies, susceptibility assays, and effectorome analysis	Syncytium initiation, vascular signalling mimicry, redox modulation, cell wall modification, and immune suppression	High
*Globodera* spp.	SPRYSEC proteins, CLE-like peptides, cell wall modifiers, defence suppressors, and variable apoplastic effectors	Effector diversity studies, resistance gene interactions, transcriptome evidence, and potato cyst nematode biology	Syncytium maintenance, host adaptation, resistance evasion, and population-level virulence variation	High to moderate
*Tylenchulus semipenetrans*	Putative nurse-cell effectors, defence suppressors, and nutrient flow regulators	Anatomical feeding-site studies, citrus pathology, and comparative effector inference	Nurse-cell conditioning, chronic feeding, and citrus slow decline pathology	Moderate to limited
*Nacobbus* spp.	Effector candidates with similarities to sedentary endoparasites and migratory stages	Transcriptome-based evidence and life-cycle comparison with root-knot and cyst nematodes	Dual strategy: early migration followed by specialised feeding-site development	Moderate
*Pratylenchus* spp.	Pectin methylesterases, endoglucanases, detoxification proteins, and candidate secreted effectors	Migratory-effector reviews, transcriptomics, and functional candidate studies	Cell wall modification, cortical migration, lesion formation, defence suppression, and repeated feeding	Moderate but expanding
*Radopholus* spp.	Pectate lyases, cell wall-degrading enzymes, and detoxification-associated candidates	Gland localisation for some enzymes, migratory endoparasite reviews, and pathology studies	Root invasion, cortical tunnelling, necrosis, and burrowing-type parasitism	Moderate
Ectoparasites and virus vectors	Feeding-associated secretions; virus retention and vector-competence traits in longidorids and trichodorids	Feeding biology, virus transmission studies, and comparative pharyngeal gland models	External cell conditioning, root-tip damage, and virus-vector facilitation in selected genera	Limited to moderate

**Table 3:** Comparative summary of oesophageal gland-derived gene product functions across nematode lifestyles, based on comparative reviews of sedentary, migratory, and ectoparasitic plant–nematode interactions [1,2,3].

Nematode Group	Representative Genera/Species	Feeding Strategy	Dominant Gland-Product Role	Host Response Induced	Parasitic Outcome
Root-knot nematodes	*Meloidogyne*	Sedentary endoparasitic	Giant-cell induction and maintenance	Multinucleate giant cells and galls	Long-term feeding and high egg production
Cyst nematodes	*Heterodera*, *Globodera*	Sedentary endoparasitic	Syncytium induction and defence suppression	Cell fusion into syncytium	Permanent nutrient sink for female development
Citrus nematode	*Tylenchulus semipenetrans*	Sedentary semi-endoparasitic	Nurse-cell establishment and chronic feeding	Specialised nurse cells in cortex	Slow decline and reduced citrus performance
False root-knot nematodes	*Nacobbus*	Migratory then sedentary	Dual migration and feeding-site effectors	Root-knot/cyst-like feeding structures	Combined migration and sedentary feeding
Root-lesion nematodes	*Pratylenchus*	Migratory endoparasitic	Cell wall degradation and repeated feeding	Cortical lesions and necrosis	Reduced root function and disease complexes
Burrowing nematodes	*Radopholus*	Migratory endoparasitic	Cortical invasion and tissue destruction	Root necrosis and tunnelling	Decline of banana, citrus and other crops
Ectoparasites	*Xiphinema*, *Longidorus*, *Trichodorus*, *Paratrichodorus*	External feeding	Cell conditioning and vector-associated feeding	Local cell damage or virus transmission	Root dysfunction and viral disease spread

**Table 4:** Transgenic, RNAi, and genome-editing approaches that have advanced oesophageal gland effector biology and nematode resistance research [18,39,41,49].

Genetic or Biotechnology Approach	Molecular Target or Product	Representative Evidence	How it Promotes This Review Subject	Main Limitation or Caution
Natural resistance genes	Plant R genes such as Mi-1.2, Hero A, Gpa-2, and Hs1pro-1	Cloned Mi-1.2 conferred root-knot nematode resistance in susceptible tomato backgrounds	Links nematode parasitism to effector recognition, defence activation, and feeding-site failure	Often species-, pathotype-, or temperature-limited; resistance can be overcome
Proteinase inhibitors and cystatins	Plant or engineered inhibitors of nematode digestive proteinases	Cystatin-based transgenic potato resistance has been tested against *Globodera pallida*	Shows that nematode feeding and nutrition can be interrupted after stylet-mediated uptake	Efficacy depends on expression level, feeding biology, and target-crop acceptance
Chemodisruptive peptides	Peptides that disrupt chemoreception or root invasion	Transgenic potatoes expressing a behaviour-disrupting peptide reduced *G. pallida* invasion in containment and field trials	Demonstrates that transgenic resistance can target invasion and host-location processes rather than killing nematodes directly	Requires precise expression and biosafety assessment
Host-delivered RNAi of effectors	Nematode effector transcripts such as 16D10 and Mi-msp2	16D10 dsRNA conferred broad root-knot resistance; Mi-msp2 RNAi reduced galls, females, and egg masses	Provides direct functional proof that gland-derived effectors are essential parasitism genes	Target selection, off-target risk, regulatory acceptance, and variable RNAi efficiency remain important
Nematode-responsive promoters	Root-, gall-, syncytium-, or infection-site inducible promoters	Recent promoter reviews emphasise infection-specific expression of RNAi, CRISPR, or killer constructs	Aligns resistance expression with the spatial and temporal biology of effector delivery	Promoter strength, specificity, and stability require validation in each crop and nematode system
Host-target manipulation	Plant genes induced or suppressed during feeding-site development	Transgenic overexpression or silencing of host genes can affect syncytium or giant-cell development	Connects nematode effectors to downstream host targets and feeding-site biology	Risk of pleiotropic effects because many host targets regulate normal development
Spray-induced or exogenous RNAi	Externally delivered dsRNA targeting nematode genes	Recent RNAi reviews emphasise nanotechnology and delivery systems for phytoparasitic nematodes	Extends effector targeting beyond stable transgenic plants and supports more flexible, non-transformative deployment	Soil stability, uptake by nematodes, formulation cost, and field persistence remain unresolved
CRISPR/Cas susceptibility gene editing	Plant compatibility or susceptibility genes exploited by nematode effectors	OsHPP04 editing enhanced rice resistance to *Meloidogyne graminicola*; MLO3 editing reduced *Rotylenchulus reniformis* reproduction in cotton	Links effector biology to plant targets and feeding-site compatibility factors	Requires careful assessment of pleiotropy, durability, regulatory status, and species specificity

**Table 5:** Practical management value of oesophageal gland effector biology, separated into diagnostic, resistance breeding, biotechnology, and integrated nematode management applications [7,9,53,54].

Management Value	Effector-Centred Use	Practical Output	Citation Support
Diagnostic and surveillance value	Use effector genes, species-specific parasitism genes and virulence-associated markers to improve detection of damaging or resistance-breaking populations.	More precise species/pathotype identification; early-warning surveillance; population-risk profiling before planting.	[7,9,55]
Resistance breeding value	Screen germplasm for failure of giant-cell, syncytium, or nurse-cell induction; use effector recognition or host-target disruption as selection logic.	Cultivars that block feeding-site initiation, reduce reproduction, or delay resistance-breaking biotypes.	[10,16,34,47]
Biotechnology value	Target essential parasitism genes using HIGS, HD-RNAi, spray-induced RNAi, nematode-responsive promoters, or CRISPR/Cas editing of plant susceptibility factors.	Effector-specific suppression of infection, feeding, development, and fecundity; reduced dependence on broad-spectrum nematicides.	[39,43,44,49]
Integrated nematode management value	Combine resistance, crop rotation, biological control, botanicals, and soil-health practices around endpoints that disrupt parasitism rather than mortality alone.	Reduced galling, lower reproduction factor, impaired feeding-site function, and improved crop performance under reduced-chemical systems.	[53,54,56]
Decision-support value	Link effector profiles to host status, host sensitivity, crop rotation, and population density data.	Risk-based crop choices, cultivar deployment, and intervention timing suited to local nematode communities.	[3,15,23]
Research-translation value	Use effector biology to design assays that measure invasion, stylet activity, feeding-site establishment, and reproduction as separate outcomes.	Mechanistic evaluation of resistant cultivars, bionematicides, and regenerative-agriculture remedies.	[7,9,26]

**Table 6:** Priority research questions and cross-cutting applications for oesophageal gland-derived gene products in plant-parasitic nematodes, as derived from the evidence gaps identified in comparative effector syntheses [3,7,9,23].

Research Domain	Priority Research Question	Suggested Approach	Expected Contribution
Root-knot nematodes (*Meloidogyne* spp.)	Which dorsal gland effectors are essential for late giant-cell maintenance and adult female fecundity?	Single-cell gland transcriptomics; effector localisation; HD-RNAi; host-target validation.	Identifies targets that disrupt reproduction after infection has already begun.
Cyst nematodes (*Heterodera* and *Globodera* spp.)	Which peptide mimics, SPRYSEC proteins and chorismate mutases determine host range and resistance-breaking?	Effector haplotyping; resistant/susceptible cultivar comparisons; host-protein interaction assays.	Improves durable resistance breeding and monitoring of virulent populations.
*Tylenchulus semipenetrans*	Which gland products induce and maintain nurse cells in citrus roots?	Gland-cell transcriptomics of female stages; nurse-cell proteomics; citrus defence profiling.	Addresses a major knowledge gap in citrus slow decline and chronic semi-endoparasitism.
*Nacobbus* spp.	How does the effector repertoire switch between migratory and sedentary phases?	Stage-specific transcriptomics; in situ hybridisation; comparative analysis with *Meloidogyne* and cyst nematodes.	Clarifies the evolutionary bridge between destructive migration and permanent feeding-site formation.
*Pratylenchus* spp.	Which enzymes and defence suppressors govern lesion formation, migration and fungal/bacterial disease complexes?	Secretome profiling; host-response time series; RNAi of cell wall and detoxification genes.	Strengthens management of lesion nematodes beyond population counts.
*Radopholus* spp.	Which pectate lyases, detoxification proteins and gland products enable burrowing and cortical necrosis?	Effector mining; banana/citrus infection models; functional knockdown and histopathology.	Supports targeted control of burrowing nematode decline syndromes.
Ectoparasitic and virus-vector nematodes	Which feeding-associated secretions condition root cells and interact with virus acquisition or delivery?	Stylet/gland proteomics; virus retention assays; feeding behaviour imaging.	Links gland biology with virus-vector epidemiology and root-damage mechanisms.
Cross-cutting crop-system application	Can effector markers improve host status, host sensitivity, and crop rotation recommendations?	Combine molecular diagnostics with ERP/CARD-type population density models and field validation.	Creates decision-support tools for sustainable and regenerative nematode management.

## Data Availability

No new experimental data were generated for this review. The synthesis is based on published literature cited in the reference list.

## Abbreviations

CEP, C-terminally encoded peptide; CLE, CLAVATA3/EMBRYO SURROUNDING REGION-related; CRISPR/Cas, clustered regularly interspaced short palindromic repeats/CRISPR-associated protein; dsRNA, double-stranded RNA; FAR, fatty-acid- and retinol-binding protein; GST, glutathione S-transferase; HD-RNAi, host-delivered RNA interference; HIGS, host-induced gene silencing; IDA, INFLORESCENCE DEFICIENT IN ABSCISSION; J2, second-stage juvenile; PSK, phytosulfokine; PSY, plant peptide containing sulfated tyrosine; RALF, rapid alkalinisation factor; RNAi, RNA interference; ROS, reactive oxygen species; SPRYSEC, SPRY-domain-containing secreted protein; VAP, venom allergen-like protein.
